# Gastrointestinal Cytoprotection by PPAR*γ* Ligands

**DOI:** 10.1155/2010/108632

**Published:** 2010-09-21

**Authors:** Yuji Naito, Tomohisa Takagi, Toshikazu Yoshikawa

**Affiliations:** Molecular Gastroenterology and Hepatology, Graduate School of Medical Science, Kyoto Prefectural University of Medicine, 465 Kajii-cho, Kawaramachi-Hirokoji, Kamigyo-ku, Kyoto 602-8566, Japan

## Abstract

Peroxisome proliferator-activated receptor *γ* (PPAR*γ*) is a nuclear receptor that is known to play a central role in lipid metabolism and insulin sensitivity as well as inflammation and cell proliferation. According to the results obtained from studies on several animal models of gastrointestinal inflammation, PPAR*γ* has been implicated in the regulation of the immune response, particularly inflammation control, and has gained importance as a potential therapeutic target in the management of gastrointestinal inflammation. In the present paper, we present the current knowledge on the role of PPAR*γ* ligands in the gastrointestinal tract.

## 1. Introduction

Peroxisome proliferator-activated receptors (PPARs) are transcription factors belonging to the nuclear receptor superfamily and have been initially described as molecular targets for compounds that cause peroxisome proliferation [1]. Thus far, 3 isotypes of PPARs (PPAR*α*, PPAR*δ* (also known as PPAR*β*), and PPAR*γ*) have been found in various species [2–5]. Of these, PPAR*γ* proved to be a key transcription factor involved in lipid metabolism and adipocyte differentiation. In addition, recent studies suggest that PPAR*γ* may be involved in the control of inflammation and especially modulation of the expression of various cytokines in monocytes and macrophages [6, 7]. Regarding the anti-inflammatory properties of PPAR*γ*, PPAR*γ* activation has been shown to antagonize the activity of activation protein-1 (AP-1), Stat 1, and nuclear factor-*κ*B (NF-*κ*B), which are known for positively controlling cytokine gene expression [6]. 

PPAR*γ* predominates the adipose tissue, large intestine, macrophages, and monocytes [6, 8–10]. Recently, it was demonstrated that 15-deoxy-Δ12, 14-prostaglandin J2 (15d-PGJ2), and various polyunsaturated fatty acids have been identified as natural receptor ligands of PPAR*γ*. In addition, thiazolidinediones such as troglitazone, pioglitazone, and rosiglitazone, which are used as antidiabetic drugs, have been developed as synthetic PPAR*γ* ligands. The use of such ligands has allowed researchers to unveil many potential roles of PPARs in pathological conditions, including atherosclerosis, inflammation, and cancer. In this paper, we present the current knowledge available on the role of PPAR*γ* in the gastrointestinal tract.

## 2. Esophagus and PPAR*γ*

Few studies have examined the role of PPAR*γ* in the esophageal mucosa. PPAR*γ* expression in the epithelium of Barrett's esophagus (BE) is elevated as compared to that in the normal esophageal squamous epithelium [11]. Reflux of gastric juice or bile acid into the esophagus causes injury to the esophageal squamous epithelium, because of which the injured esophageal mucosa is replaced by columnar epithelium; this entity is called BE. Importantly, BE is the major risk factor for esophageal adenocarcinoma. The PPAR*γ* ligands pioglitazone and ciglitazone when used alone inhibited cell proliferation in OE33 cells derived from esophageal adenocarcinoma [11, 12]; this result suggests that PPAR*γ* plays an important role in Barrett's carcinogenesis and that PPAR*γ* ligands may be useful as new therapeutic agents for the prevention and treatment of Barrett's carcinoma. However, because it has been reported that OE33-derived transplantable adenocarcinoma was enhanced in vivo by systemic PPAR*γ* activation due to cell proliferation, the detailed role of PPAR*γ* in the esophagus remains controversial [11]. 

In regard to human esophageal squamous cell carcinoma (SCC), PPAR*γ* has been found to be expressed in human SCC cell lines such as TE-1, TE-2, TE-5, TE-7, TE-8, TE-9, and TE-10 [13, 14]. Interestingly, PPAR*γ* ligands such as 15-deoxy-Δ12,14-prostaglandin J2 (15d-PGJ2), and troglitazone significantly inhibited the proliferation of these SCC cells in a dose-dependent manner [13]. On the other hand, Terashita et al. reported that although PPAR*γ* mRNA expression was detectable in the majority of human SCC tissues and all the normal esophageal mucosa, PPAR*γ* mRNA expression level was significantly decreased in SCC tissues compared to normal esophageal mucosa [14]. In their clinicopathological studies, PPAR*γ* mRNA expression level in the patients with esophageal SCC with extensive lymph node metastasis was significantly decreased compared with those with less extensive lymph node metastasis. Thus, the role of PPAR*γ* remains controversial in esophageal SCC as well as esophageal adenocarcinoma, and further examinations is required to gain a better understanding of the role of PPAR*γ* in esophageal tumors. 

## 3. Stomach and PPAR*γ*

**Figure 1:**
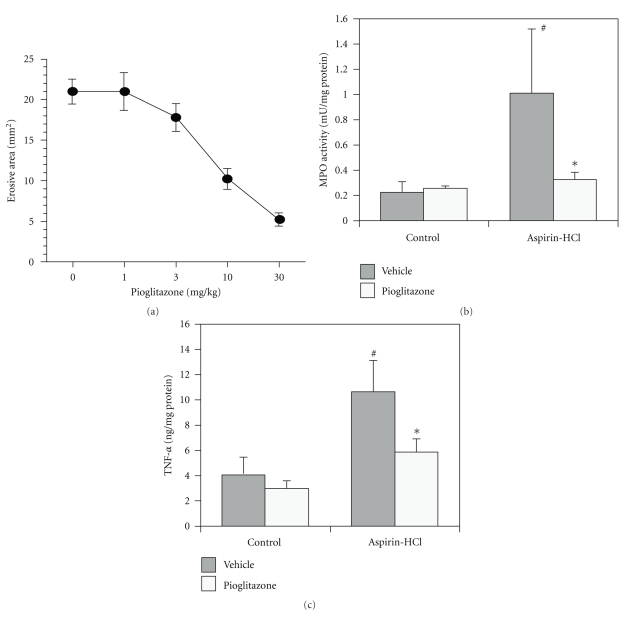
Effect of increasing doses of pioglitazone on acute gastric mucosal injury induced by aspirin-HCl in rats (a) The effect of pioglitazone on tissue-associated myeloperoxidase (MPO) activity (b) and TNF-*α* content (c) induced by aspirin-HCl in the gastric mucosa. TNF-*α* content and MPO activity in the gastric mucosa increased after aspirin administration. This increase in TNF-*α* content and MPO activity was inhibited by pioglitazone treatment.

In several studies, it has been demonstrated that PPAR*γ* ligands reduced the extent of mucosal damage and inhibited the inflammatory response to gastric inflammation (Table 1). First, we demonstrated that pioglitazone, a specific PPAR*γ* ligand, ameliorated aspirin-induced injury to the gastric mucosa in rats (Figure 1) and inhibited the increase in neutrophil accumulation associated with gastric mucosal TNF-*α* contents, which were measured by Enzyme-Linked Immunosorbent Assay (ELISA) [15]. PPAR*γ* has also been implicated in the control of gastric mucosal damage induced by ischemia-reperfusion injury [16]. Pioglitazone, rosiglitazone, troglitazone, and 15d-PGJ2 inhibited gastric mucosal damage induced by ischemia-reperfusion injury through the inhibition of cytokines expression such as TNF-*α* and IL-1*β*, and the inhibition of the neutrophil accumulation in the gastric mucosa [16–21]. Interestingly, regarding the expression of intercellular adhesion molecule-1 (ICAM-1), which played an important role in neutrophil infiltration into gastric mucosa, the increased expression of ICAM-1 after gastric ischemia reperfusion was also inhibited by treatment with these PPAR*γ* ligands [18, 21]. Thus, PPAR*γ* mediated the amelioration of the inflammatory responses involved in acute gastric damage. 

In gastric ulcer healing, it seems that the activation of PPAR*γ* ligands produces favorable effects. Pioglitazone accelerates the healing of acetic acid-induced gastric ulcers by the triggering anti-inflammatory effects, including the suppression of interleukin (IL)-1*β*, tumor necrosis factor-*α* (TNF-*α*, cyclooxygenase (COX)-2, and inducible nitric oxide synthase (iNOS), and by increasing the expression of heat shock protein 70 (HSP70) [23]. Brzozowski et al. also demonstrated that pioglitazone accelerates the healing of gastric ulcers induced by topical application of 100% ethanol or water immersion and restraint stress [24]. In addition to suppression of the proinflammatory cytokines TNF-*α* and interleukin-1*β* (IL-1*β*, pioglitazone enhanced angiogenesis through increased expression of platelet endothelial cell adhesion molecule-1 (PECAM-1)). Furthermore, Lahiri et al. also reported that pioglitazone-induced activation of PPAR*γ* mediated gastric ulcer healing in rats, and this pioglitazone-mediated gastroprotective effect is also involved in glucocorticoid receptor activation during chronic gastric ulcer healing [22]. Hence, together the data suggest that PPAR*γ* is a novel therapeutic target molecule and PPAR*γ* ligands can be used as therapeutic agents for gastric ulcerative lesion.

Interestingly, PPAR*γ* plays a crucial role in gastric mucosal injury in relation to *H. pylori* (*Helicobacter pylori)* infection. As It has been well known that *Helicobacter pylori* infection plays important role as the cause of chronic gastritis [37] and as a definite carcinogen in gastric cancer [38], understanding how PPAR*γ* is involved in *H. pylori* infection may lead to the development of therapeutic strategy for *H. pylori* infection. B. L. Slomiany and A. Slomiany have demonstrated that *H. pylori *lipopolysaccharide- (LPS-) elicited mucosal inflammatory responses were accompanied by a massive epithelial cell apoptosis, upregulation of iNOS, and COX-2 expression, and PPAR*γ* ligand ciglitazone suppresses these gastric mucosal inflammatory responses and may provide therapeutic benefits such as the amelioration of inflammation associated with *H. pylori* infection [39]. In fact, PPAR*γ* expression in the gastric mucosa increases with *H. pylori* infection and produces cytoprotective and anti-inflammatory effects in the gastric mucosa [40]. Furthermore, Konturek et al. also have shown that PPAR*γ* is implicated in *H. pylori*-related gastric carcinogenesis and that PPAR*γ* agonists may have a therapeutic role in cancer [41]. On experimental investigation, it was found that PPAR*γ* suppresses gastric carcinogenesis and that PPAR*γ* ligands such as troglitazone and ciglitazone are potential agents for gastric carcinoma because they inhibit PPAR*γ*-dependant cell proliferation [42–44]. 

On the other hand, the importance of PPAR*γ* polymorphism (Pro12Ala) has been reported. The PPAR*γ* Pro12Ala polymorphism has been reported to show decreased binding to the promoter element and demonstrates weaker transactivation of responsive promoters [45]. It has been reported that PPAR*γ* polymorphism (Pro12Ala) is associated with various disease including diabetes, asthma, endometriosis, polycystic ovary, and colorectal cancer [46–50]. Regarding to gastric disease, this PPAR*γ* polymorphism is associated with not only gastric ulcer but also gastric adenocarcinoma [51–53].

## 4. Intestine and PPAR*γ*

In many studies, PPAR*γ* has been reported to play a role in the small and large intestine. This is probably because of high PPAR*γ* expression in the colon tissue. The high expression of PPAR*γ* seems to be related to intestinal bacteria. Dubuquoy et al. showed that PPAR*γ* expression in the colon tissue was greater in conventional mice than in germ-free mice [54]. More interestingly, they demonstrated that PPAR*γ* expression was weaker in the colon tissue of mice deleted for the Toll-like receptor (TLR4) than in that of wild-type mice. Furthermore, in colonic epithelial cells such as HT-29 and Caco-2, PPAR*γ* expression was markedly increased because of the presence of LPSs [55]. These data indicate that the role of bacteria-derived LPS in the regulation of PPAR expression is more crucial in the colon tissue than in other parts of the gastrointestinal tract. 

With regard to the anti-inflammatory properties of P PPAR*γ* in intestinal inflammation, the therapeutic efficacy of PPAR*γ* ligands has been evaluated in various different models of intestinal inflammation (Table 2). To determine the role of PPAR*γ* in intestinal ischemia-reperfusion injury, Nakajima et al. [18] used PPAR*γ*-deficient mice and the PPAR*γ* agonist rosiglitazone. They demonstrated the dramatic protective effects of rosiglitazone on both local and remote organ injury after intestinal ischemia-reperfusion injury and showed that the endogenous absence of PPAR*γ* leads to aggravated injury in this model. In several studies, it has been demonstrated that the activation of PPAR*γ* by PPAR*γ* ligands inhibited intestinal ischemia-reperfusion injury [25, 26, 56]. One possible mechanism by which PPAR*γ* activation helps in protection against ischemia-reperfusion injury is through the inhibition of NF-*κ*B-mediated transcription. The inhibition of NF-*κ*B activation was confirmed by several approaches, including electrophoretic mobility shift assays, immunohistochemistry using a phosphorylation state-specific antibody for I*κ*B, and mRNA levels of TNF-*α* and intercellular adhesion molecule-1 (ICAM-1), which are downstream targets of NF-*κ*B.

**Figure 2:**
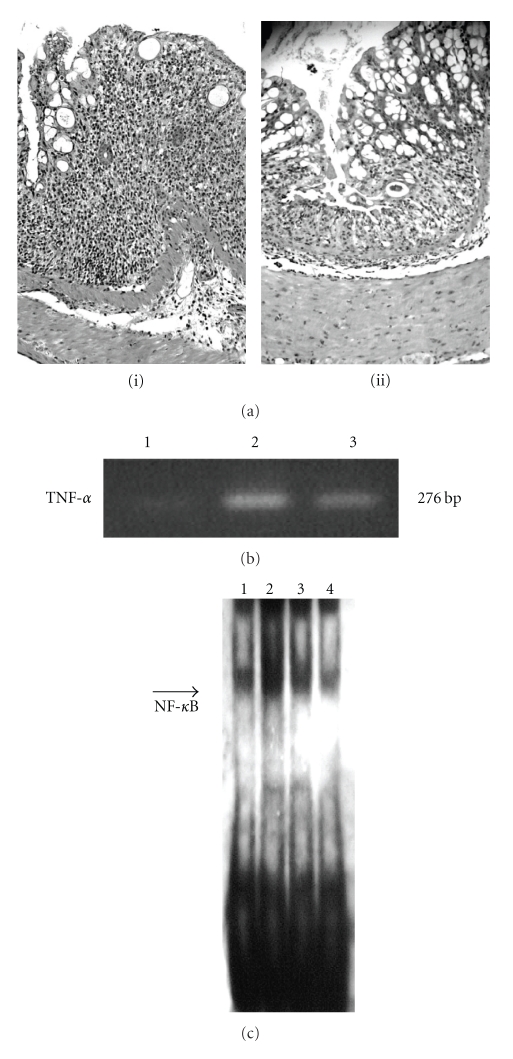
(a) Image showing the appearance of the colon in a mouse that was administered dextran sulfate sodium (DSS) (i) and pioglitazone (ii). Loss and shortening of crypts, mucosal erosions, inflammatory cell infiltration, and goblet cell depletion are seen in (i). In (ii), smaller erosions are associated with less inflammatory cell infiltration. Hematoxylin and eosin staining, ×10. Effects of pioglitazone on mRNA expression of TNF-*α* (b) and on DNA-binding activity of NF-*κ*B (c) in colonic tissues of mice that were administered DSS. Reverse transcriptase-polymerase chain reaction (RT-PCR), electrophoresis mobility shift assay (EMSA) of sham-operated colon (lane 1), DSS-induced inflamed tissue (lane 2), colon treated with 3 mg/kg pioglitazone (lane 3), and sham-operated colon treated with pioglitazone (lane 4). TNF-*α* mRNA and NF-*κ*B DNA-binding activity were upregulated in inflamed colonic tissue (lane 2); this upregulation was suppressed by pioglitazone administration (lane 3).

Inflammatory bowel diseases (IBDs) such as ulcerative colitis (UC) and Crohn's disease (CD) constitute chronic and recurrent intestinal inflammatory disorders; the precise pathogenesis of these disorders remains unknown [57]. Therefore, it is very important to identify novel therapeutic molecules for IBDs. In this regard, PPAR*γ* may be a novel therapeutic target. Su et al. showed that PPAR*γ* ligands markedly reduced colonic inflammation in a mouse model of IBD [27]. We also reported that pioglitazone had a protective effect against murine dextran sulfate sodium- (DSS-) induced colitis; a model of colitis induced in this manner is commonly used as a UC model in association with inhibition of the NF-*κ*B-cytokine cascade [29] (Figure 2). In mice, overexpression of PPAR*γ* by an adenoviral construct in mucosal epithelial cells was associated with amelioration of experimental inflammation [58], and this study supports the hypothesis that the upregulation of PPAR*γ* expression itself may have a protective effect against colitis. In another study, in which colitis was induced by trinitrobenzene sulfonic acid (TNBS) and used as a CD model, PPAR*γ* ligands such as pioglitazone [30], rosiglitazone [33], and troglitazone [32] inhibited the development of the intestinal inflammation. 

DSS-induced and TNBS-induced colitis are widely used models of chemically induced intestinal inflammation. In studies on immune-reactive cells in the intestinal tissue of UC and CD patients, it has been demonstrated that the deregulated immune response plays a crucial role in the onset of IBD. Therefore, other types of colitis models are widely used, including a transfer colitis model produced by transfer of a T-cell population (CD4^+^CD45RBhigh T cells) that lacks regulatory cells into an immunodeficient host, spontaneous colitis model such as the SAMP/Yit mouse, and genetic colitis model such as interleukin IL-10-deficient mice. In a previous study, it was found that rosiglitazone delayed the onset of colitis in IL-10-deficient mice [35]. Further, it was also found that crypt hyperplasia, caused by increased mitotic activity of crypt epithelial cells, was also delayed by rosiglitazone accompanied by the decreased expression of interferon-*γ* (IFN-*γ*), IL-17, TNF-*α*, and iNOS in the colon. Sugawara et al. have identified PPAR*γ* as a CD susceptibility gene in both mice and humans [36]. The administration of rosiglitazone inhibited SAMP/Yit ileitis through regulation of PPAR*γ* activity in the crypts of the small intestine.

With regard to the relation between immune cells and PPAR*γ*, it has been reported that PPAR*γ* ligands modulate dendritic cell (DC) function to elicit the development of anergic CD4^+^ T cells [59]. Hontecillas and Bassaganya-Riera demonstrated that effector CD4^+^ cell function was downregulated by activated regulatory T cells (Tregs), which were activated by endogenously produced PPAR*γ* [60]. In fact, they also showed that PPAR*γ* deficiency in Tregs impairs the ability of Tregs to prevent T-cell transfer-induced colitis. With regard to the CD4^+^ transfer colitis model, Bassaganya-Riera et al. showed that conjugated linoleic acid ameliorated colitis [31]. 

Thus, PPAR*γ* ligands reduced mucosal damage and prevented or downregulated the inflammatory response in several murine models of intestinal inflammation. These anti-inflammatory effects suggest that PPAR*γ* agonists may provide a novel therapeutic approach for treating IBD. In fact, rosiglitazone produced beneficial effects in the treatment of UC in an open-label trial [61]. In this study, rosiglitazone treatment for UC patients refractory to conventional treatment yielded a decrease in disease activity index score. Although the results of this pilot study are yet to be confirmed, PPAR*γ* ligands may be novel therapeutic agents for treating IBD.

More interestingly, Rousseaux et al. showed that the therapeutic effect of 5-aminosalicylic acid (5-ASA) may be mediated by PPAR*γ* [34]. Heterozygous PPAR*γ*-knockout mice were refractory to 5-ASA treatment, and 5-ASA directly induced PPAR*γ* expression in colonic epithelial cells in vitro. Although 5-ASA is one of the conventional agents uses for IBD treatment, the precise mechanism underlying the protective effect of 5-ASA remained unclear. These data reveal that PPAR*γ* is a target of 5-ASA; this finding underlies the anti-inflammatory effects produced in the colon. 

Many studies have investigated the relation between PPAR*γ* and colon cancer. PPAR*γ* is expressed at high levels in primary colon tumors and colon cancer cell lines [62]. On the other hand, PPAR*γ* ligands cause withdrawal of colon cancer cell lines from the cell cycle, inhibit cell growth, and promote differentiation [63, 64]. Based on these finding, it appears as if PPAR*γ* may be exerting some other actions rather than regulating tumor growth. One possibility is that PPAR*γ* expression by the tumor may program these cells to be less immunogenic or possibly lead to the secretion of molecules that would end up promoting tumor growth. Osawa et al. recently showed that continuous feeding of pioglitazone reduced the aberrant crypt foci formation and notably suppressed colon tumors [65]. Although there is a contradictory study in which APC^min /+^ mice showed an increased number of polyps when subjected to a PPAR*γ* agonist [66], many research studies have shown that PPAR*γ* agonists seem to have inhibitory effects on the proliferation of colon cancer cells. PPAR*γ* ligands may represent a new group of biological agents that can be used for the management of colon cancer.

## 5. Conclusion

In this paper, we focused on the therapeutic effect of PPAR*γ* agonists in gastrointestinal inflammation. We performed studies using several animal models of gastrointestinal inflammation and accumulated evidence suggesting that PPAR*γ* plays a crucial role in gastrointestinal inflammation. It was found that PPAR*γ* ligand therapy reduced a wide variety of inflammatory indices in different animal models, but the underlying mechanism by which PPAR*γ* activation produces these effects was not fully established. We expect that the precise mechanism by which PPAR*γ* ligands produce anti-inflammatory properties will be clarified in the near future.

## Figures and Tables

**Table 1:** Cytoprotective properties of *P* *P* *A* *R* *γ* in experimental model of gastric injuries.

Model	PPAR*γ* ligand	References
Gastric Ulcer		
(Acute gastric damage)	Pioglitazone	Naito et al. [15]
(*H. pylori*-induced gastritis)	Citiglitazone	B. L. Slomiany and A. Slomiany [39]
(Gastric ulcer Healing)	Pioglitazone	Konturek et al. [17], Brzozowski et al. [24], Lahiri et al. [22]
		
Ischemia-reperfusion	Pioglitazone	Ichikawa et al. [16], Konturek et al. [23], Wada et al. [21]
	Rosiglitazone	Villegas et al. [20], Wada et al. [21]
	Troglitazone	Wada et al. [21]
	15d-PGJ2	Takagi et al. [19]

**Table 2:** Cytoprotective properties of PPAR*γ* in experimental model of the intestinal inflammation.

Model	PPAR*γ* ligand	References
Ischemia/reperfusion injury	Rosiglitazone	Nakajima et al. [18]
	Pioglitazone	Naito et al. [25]
	15d-PGJ2	Cuzzocrea et al. [26]
		
DSS colitis	Troglitazone	Su et al. [27]
	Rosiglitazone	Saubemann et al. [28]
	Pioglitazone	Takagi et al. [29], Schaefer et al. [30]
	CLA	Bassaganya-Riera et al. [31]
		
TNBS colitis	Troglitazone	Desreumaux et al. [32]
	Rosiglitazone	Sánchez-Hidalgo et al. [33]
	Pioglitazone	Schaefer et al. [30]
	5-ASA	Rousseaux et al. [34]
		
CD4+CD45RBhigh (transfer colitis model)	CLA	Bassaganya-Riera et al. [31]
IL-10 KO (genetic colitis model)	Rosiglitazone	Lytle et al. [35]
SAMP1/YitFC (spontenouse colitis model)	Rosiglitazone	Sugawara et al. [36]

DSS, dextran sodium sulphate; TNBS, 2,4,6-trinitrobenzene sulfonic acid; 15dPGJ2, 15-deoxy-D12,14-prostaglandin J2; CLA, conjugated linoleic acid; 5-ASA, 5-aminosalycilic acid; IL-10 KO, interleukin 10 knockout mice.
